# Retraction: Evaluation of all-for-one tourism development level: Evidence from Xinjiang production and construction corps, China

**DOI:** 10.1371/journal.pone.0351196

**Published:** 2026-06-23

**Authors:** The PLOS One Editors

The *PLOS One* Editors retract this article [1] due to concerns about compromised peer review and compliance with PLOS policy on Manipulation of the Publication Process.

All authors did not agree with the retraction.
